# Chromatin marks and ambient temperature-dependent flowering strike up a novel liaison

**DOI:** 10.1186/s13059-017-1259-2

**Published:** 2017-06-19

**Authors:** Alexander Steffen, Dorothee Staiger

**Affiliations:** Molecular Cell Physiology, Faculty of Biology, Bielefeld University, Bielefeld, Germany

## Abstract

A distinct chromatin mark, H3K36me3, has been found to engage in temperature-dependent alternative splicing and ambient temperature-dependent flowering-time control in *Arabidopsis*.

As sessile organisms plants need to adapt precisely to changing environments in order to ensure reproductive success. Environmental temperature in particular influences vegetative development and the transition to flowering. On a molecular level, alternative splicing of mRNAs is a major plant response to changes in ambient temperature. A recent publication shows that a particular chromatin mark, histone H3 lysine 36 trimethylation (H3K36me3), affects temperature-dependent alternative splicing and the regulation of flowering time by ambient temperature [1].

Alternative pre-mRNA splicing is a molecular mechanism that produces different mRNA isoforms from one pre-mRNA. These isoforms can harbor different *cis*-regulatory motifs, such as interaction sites for RNA-binding proteins or microRNAs, leading to altered transcript stability and, ultimately, variation in the transcriptome. In the extreme form of pre-mRNA splicing, transcript isoforms that have a premature termination codon can be recognized as “aberrant” and funneled into the nonsense-mediated decay pathway. Furthermore, a variable combination of exons provides blueprints for proteins that have distinct domain compositions, increasing the complexity of the proteome.

Pre-mRNA splicing is accomplished by the spliceosome, a megaparticle comprising RNAs and proteins that assembles at each intron. Since the observation that different promoters affect splicing outcome, it has become obvious that splicing is initiated during transcription, while the RNA is still associated with chromatin, in a process dubbed “cotranscriptional” splicing [2]. Thus, transcriptional dynamics regulate the interaction of the spliceosome and pre-mRNAs. Consequently, the chromatin state not only determines which part of the genome is actively transcribed but also how pre-mRNA splicing generates mature mRNAs. An important factor in the regulation of chromatin state is the posttranslational modification of the histones that make up the nucleosomes. The dynamics of chromatin marks are governed by “writers” that deposit the mark and by “erasers” that remove it. The marks are interpreted by “readers” that bind to the chromatin mark and recruit other factors.

The impact of chromatin state on splicing in plants is not known, but ample evidence exists in higher eukaryotes. For example, in mammals, H3K36me3 is enriched in exons and regulates exon skipping [2]. In their article, Pajoro and colleagues [1] hypothesized that H3K36me3 may affect splicing outcome in *Arabidopsis* too. In early studies of alternative splicing in plants, both heat shock and exposure to low temperature were found to cause changes in the splicing patterns of a suite of genes. Not only massive temperature changes but also ambient temperature changes of ±4 °C affect splicing in *Arabidopsis* [3]. Furthermore, alternative splicing of flowering time genes was observed when plants were transferred from 16 to 25 °C, a change that accelerates flowering [4]. Therefore, Pajoro and colleagues set out to test a possible connection between H3K36me3 and ambient temperature-dependent splicing [1].

## Ambient temperature affects alternative splicing

In their recent work, Pajoro and colleagues addressed the molecular underpinnings of ambient temperature-dependent flowering by combining a global alternative splicing analysis by transcriptome sequencing with chromatin immunoprecipitation sequencing (ChIP-seq) analysis of histone methylation pattern [1]. *Arabidopsis* plants were grown at 16 °C for 5 weeks in short days and then shifted to 25 °C to induce the transition to flowering by the ambient temperature pathway. Plants were sampled after 1, 3, and 5 days to monitor the onset, persistence, and a potential reversion of transcriptome changes.

Around 700 splicing events changed in response to increasing temperature. The majority of changes were already detectable after one day and remained stable for the experimental timespan. Among the affected genes was the key flowering-time regulator *FLOWERING LOCUS M* (*FLM*). Alternative splicing of *FLM* has been shown to generate different protein variants at increasing temperatures, leading to transcriptional upregulation of the flower-promoting factor *FT* at elevated temperatures [5, 6]. Moreover, the splicing patterns of transcripts encoding serine-arginine (SR)-rich splicing factors or the subunits of the U2 snRNP auxiliary factor changed in response to rising temperature. Alternative splicing of splicing regulators can lead to the production of factors with different activities, and thus can coordinately regulate cohorts of downstream targets.

## A causal link between H3K36me3 and ambient temperature-dependent flowering

To correlate changes in alternative splicing patterns with changes at the chromatin level, Pajoro and colleagues [1] focused on H3K36me3. A global determination of H3K36me3 by ChIP-seq at 16 °C and one day after increasing the temperature to 25 °C revealed around 60,000 regions in which H3K36me3 was enriched. This mark was found in 96% of the genes that undergo differential splicing upon increasing temperature, whereas it was present in only 65% of the genes changing in steady-state in response to the temperature change. Importantly, the authors observed changes in H3K36me3 deposition in the differentially spliced genes between tissue harvested at 16 and 25 °C, respectively. Overall, higher levels of the H3K36me3 mark were observed in a region around 500 nucleotides downstream of transcription start sites after the switch to 25 °C. This overrepresentation of the H3K36me3 mark in plants switched to warmer conditions implicates H3K36me3 in the regulation of temperature-induced alternative splicing.

Next, the authors set out to causally link deposition of H3K36me3 marks with temperature-dependent splicing. They made use of mutants that have defective SDG8 and SDG26 methyltransferases, and thus reduced H3K36 methylation. Global alternative splicing analysis showed that when shifted to 25 °C, temperature-dependent alternative splicing events in these mutants, including the splicing of flowering-time genes such as *FLM*, are changed relative to those in wild-type plants. At 16 °C, only small differences between wild-type plants and the mutants were observed, pointing to a specific role of H3K36me3 in ambient temperature-dependent splicing, rather than to a role of this mark for splicing in general.

**Fig. 1:**
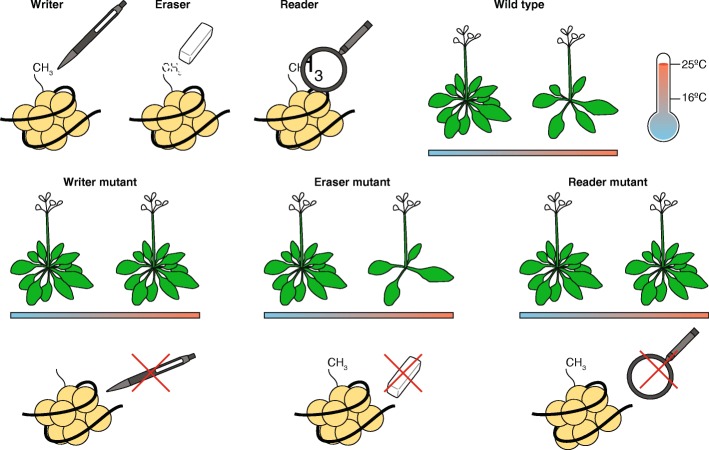
Mutations in writers, erasers, or readers of the H3K36me3 mark affect the acceleration of flowering time by increasing ambient temperature. In wild-type plants, flowering is accelerated upon a shift to warmer temperatures. Writer mutants that are impaired in deposition of the H3K36me3 mark do not show accelerated flowering in warm conditions, i.e., they flower with more or less the same number of leaves at 16 and 25 °C. In contrast, in an eraser mutant impaired in removal of the chromatin mark, acceleration of flowering by increasing temperature is greater than in wild-type plants. A reader mutant that does not recognize H3K36me3 will not react to elevated temperatures with acceleration of flowering. Acceleration of flowering time is reflective of a shorter vegetative growth period, reflected here by a decreased number of rosette leaves

Pajoro and colleagues determined the functional consequences of H3K36me3-dependent alternative splicing for flowering time, making use of writer, eraser, and reader mutants (Fig. 1). Indeed, the methyltransferase mutants *sdg8-2* and *sdg26-1* flowered at more or less the same time irrespective of ambient temperature [1]. For plants with aberrant levels of an eraser—the H3K36 demethylase mutant *jmj30-1*—acceleration of flowering by higher temperatures was somewhat stronger than in wild-type plants, whereas JMJ30-overexpressing plants had reduced histone methylation and very limited acceleration in flowering time upon a shift to higher temperatures. Furthermore, the *mrg1 mrg2* mutant, which is defective in the two potential “readers” MORF RELATED GENE 1 (MRG1) and MRG2, showed delayed temperature-induced flowering. In mammals, the related MRG15 protein binds to the H3K36me3 mark and recruits POLYPYRIMIDINE TRACT BINDING PROTEIN, which interacts with the pyrimidine-rich region at 3′ splice sites, thus serving as an adapter between chromatin and the splicing machinery [7].

## Conclusions

For the first time, a connection between a particular histone mark, H3K36me3, and ambient temperature-dependent alternative splicing has been found in *Arabidopsis*. Although there are differences in alternative splicing between animals and plants, the impact of H3K36me3 is shared.

The observation that plants that have altered levels of H3K36me3 writers, readers, and erasers are impaired in temperature-induced flowering establishes a crucial role of H3K36me3 in the ambient temperature-dependent flowering pathway. Flowering is accelerated not only by changes in temperature within the physiological range, but also by prolonged exposure to low temperatures, known as vernalization. During the cold period, a steady increase of the repressive histone mark H3K27me3 at *FLOWERING LOCUS C* (*FLC*) leads to downregulation of this prominent floral repressor, enabling the plant to flower upon the return of a warmer season [8]. Here, Pajoro and colleagues reveal another crucial role of chromatin modification in another temperature-dependent flowering pathway. Flowering at warmer temperatures involves changes in alternative splicing through deposition of H3K36me3 rather than epigenetic silencing of a floral repressor at colder temperatures. Given that these modifications are stable, they could provide a memory of ambient temperature throughout the life cycle of the plant. In this respect, it is interesting to note that the histone demethylase JMJ30 and its homologue JMJ32 have also been implicated in preventing precocious flowering at elevated temperatures by removing the repressive H3K27me3 mark at the *FLC* locus [9].

## Outlook

Pajoro and colleagues have found that changes in alternative splicing are manifested one day following an increase in temperature. As changes in chromatin marks serve to adjust the transcriptome dynamically “on short notice”, it would be worthwhile to perform a similar analysis with a higher temporal resolution to monitor earlier events. Furthermore, it would be interesting to examine whether there is a reciprocal effect of splicing on the chromatin state, because splicing has been shown to enhance the recruitment of a methyltransferase and the methylation of H3K36 [10].

## Abbreviations

ChIP-seq: Chromatin immunoprecipitation sequencing
FLM: FLOWERING LOCUS M
H3K36me3: Histone H3 lysine 36 trimethylation
MRG1: MORF RELATED GENE 1
